# Field-testing a psychosocial assessment scoring form for TMD patients - summarizing axis II instruments

**DOI:** 10.1186/s12903-020-01248-7

**Published:** 2020-10-01

**Authors:** Tamara Günther, Oliver Schierz, Sebastian Hahnel, Angelika Rauch

**Affiliations:** Department of Prosthodontics and Materials Science, University of Leipzig, Liebigstr. 12, 04103, Leipzig, Germany

**Keywords:** Behavioral sciences, Craniomandibular disorders, Depression, Psychological

## Abstract

**Background:**

The etiology of temporomandibular disorders (TMD) can be explained on the basis of a biopsychosocial model. However, psychosocial assessment is challenging in daily dental practice. The purpose of the current study was to field-test the practicability of a novel psychosocial assessment scoring form regarding the reliability of scoring procedures and the opinion of examiners. The working hypotheses were that the scoring results of inexperienced undergraduate students were similar to the results collected by a gold standard and that the scoring form was easy to use.

**Methods:**

A psychosocial assessment scoring form was developed in accordance with guidelines of the Diagnostic Criteria for Temporomandibular Disorders (DC/TMD), including results of the Graded Chronic Pain Scale (GCPS), Patient Health Questionnaires (PHQ), and Generalized Anxiety Disorders (GAD). Inexperienced operators (undergraduate students) examined patients with TMD-associated complaints and rated the practicability of the scoring form. The scoring results were recalculated by two experienced operators and a consensus was defined as a gold standard. Reliability coefficients were determined comparing results of the gold standard and the inexperienced operators.

**Results:**

Sixty-five examiners used the scoring form to obtain results for patients with TMD-associated complaints. Of the patients, 78.8% received a diagnosis according to DC/TMD decision trees. Two-thirds of the operators (62.9%) stated that the form was easy to complete, and 83.0% would use it in their dental practice. The reliability coefficients ranged between 0.81–1.00.

**Conclusions:**

Within the limitations of the present study, the psychosocial assessment scoring form seems to be an easy-to-use and practicable tool. The vast majority of the inexperienced examiners recommended the application.

## Background

Temporomandibular disorders (TMD) have a prevalence of 10% in the general population [1]. TMD is not solely caused by somatic matters but can have overlapping psychological and social comorbidities [2, 3]. As a result, TMD pain is explained by a biopsychosocial model that is strongly influenced by both physical (axis I) and psychosocial aspects (axis II) [4].

Psychosocial factors in patients with TMD mostly include anxiety, depression, and social variables, such as home or work environment [5, 6]. Various research groups demonstrated that non-specific physical symptoms (somatization) and depression play important roles in the etiology and treatment of TMD. In patients with TMD, the prevalence of non-specific physical symptoms is estimated to range from 28.5–76.6%, and moderate-to-severe levels of depression have a prevalence from 21.4–60.1% [7]. These comorbidities might have a negative effect on the treatment of TMD [8], while anxiety seems to be a less important factor in the etiology of TMD [9, 10]. The grading of chronicity might be used to tailor specific treatment regimens that address individual psychosocial adaption [11].

In most countries, general dentists are mainly involved in the care and treatment of patients with TMD. For this reason, it is particularly surprising that a recent systematic review emphasized that psychosocial aspects are regularly neglected in the education of dentists and dental hygienists, demanding an urgent need for improvements. The authors recommended easy-to-use, reliable, and validated tools for the assessment of psychological comorbidities in education and dental practice [5].

In 2014, the Diagnostic Criteria for Temporomandibular Disorders (DC/TMD) were published and contained guidelines for the physical examination and psychosocial assessment of patients with TMD [12, 13]. The latter is accompanied by a paper-based, scoring report form that summarizes the axis II results of the self-report questionnaires (Table 1) within categories. These categories can be arranged as a “green, yellow and red flag” system. Since these questionnaires are recommended even in general medicine [20, 21], the scoring form for psychosocial assessment seems to be an interesting and systematic tool for implementing psychosocial assessments procedures into dental education and clinical practice.

**Table 1:** Recommended axis II assessment instruments by Diagnostic Criteria for Temporomandibular Disorders (DC/TMD)

Instrument	Abbreviation	Number of items	Description
Graded Chronic Pain Scale [14]	GCPS 2.0	8	Grades the severity of chronic pain
Patient Health Questionnaire-9 [15]	PHQ-9	9	Screening for depression
Patient Health Questionnaire-15 [16]	PHQ-15	15	Screening for non-specific physical symptoms (somatization)
Generalized Anxiety Disorder-7 [17]	GAD-7	7	Screening for anxiety
Oral Behaviors Checklist [18]	OBC	21	Screening for parafunctions
Jaw Functional Limitation Scale [19]	JFLS-8 JFLS-20	8 short form 20 long form	Screening for functional limitations
Pain drawing	–	Mouth and teeth, Head (right and left), Body (front and back)	Pain location and spreading of pain

Thus, the aim of the current investigation was to field-test a newly developed psychosocial assessment scoring form to assess the reliability of the scoring procedures and the opinion of inexperienced examiners. The working hypotheses were that the scoring results of undergraduate students were similar to those of a gold standard and that the scoring form was easy for inexperienced undergraduate students to use.

## Methods

A scoring form for psychosocial assessment was developed in accordance with the cut-off values presented in the self-report instrument manual issued by the DC/TMD [13]. The scoring form was pilot-tested by fifteen inexperienced operators (undergraduate students; mean age 24 ± 1 years; age range 22–31 years; 73.3% females) who had never before examined the psychosocial characteristics of a patient. By using the psychosocial instruments recommended by DC/TMD, each undergraduate assessed one TMD patient for axis II features in accordance with the DC/TMD guidelines, including GCPS (1-month version), PHQ-9, PHQ-15, and GAD-7 (Table 1). As population-based norms for the OBC, JFLS, and pain drawing are not yet established, cut-off values of these instruments were not included in the psychosocial assessment scoring form. The patients completed the psychosocial questionnaires at home, and the forms were finally evaluated by the undergraduate students in a university setting. The undergraduate students were asked to describe problems they encountered during the application of the psychosocial assessment scoring form.

**Fig. 1:**
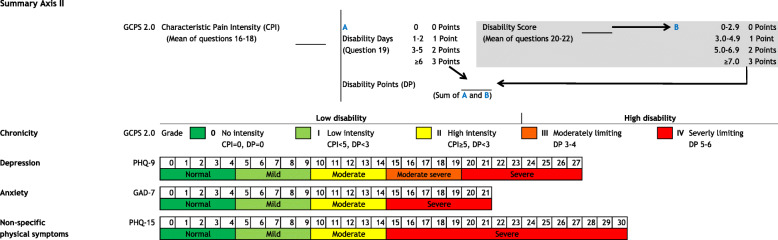
Modified psychosocial assessment scoring form for axis II instruments according to the Diagnostic Criteria for Temporomandibular Disorders (DC/TMD) used in dental undergraduate courses; English version

The scoring form was subsequently modified, taking the comments of the undergraduate students into consideration (Fig. 1). Field-testing was performed within three consecutive clinical undergraduate courses in prosthetic dentistry between 2018 and 2019. In parallel to the courses, a series of compulsory lectures focusing on TMD patients, including education regarding the physical and psychosocial aspects of these patients, was offered. In the clinical courses, patients with TMD-associated symptoms were examined for psychosocial characteristics and physical TMD signs. Each undergraduate examined one patient to assess psychosocial characteristics for the first time and in accordance with the procedure described in the pilot-testing. Finally, the undergraduate students completed a questionnaire with a 5-point Likert scale (strongly agree, agree, undecided, disagree, strongly disagree). The statements to be answered were formulated as follows: “It was easy for me to evaluate psychosocial characteristics by completing the scoring report form,” and “I would implement the psychosocial scoring form for treating patients in my future dental practice.” A physical examination was performed by each student utilizing the DC/TMD examination form; the DC/TMD decision trees were used to establish a diagnosis.

All the patients included in this study were consecutively recruited from among patients with TMD-associated complaints who received treatment in the clinical undergraduate courses. Those patients are referred within the dental school from other divisions or from general practitioners in the surrounding area. As our dental school specializes in the diagnosis and treatment of and education about TMD, every undergraduate student has to treat at least one patient with TMD-associated complaints during each of two clinical courses in prosthodontics. The conventional workflow is: following collection and analysis of the axis I and axis II findings by the student, all the outcomes are evaluated one-to-one with a supervisor. The student discusses moderate psychosocial characteristics with the patient and is guided by an expert in TMD (DC/TMD Level 3). Patients with conspicuous results, such as a pain-related disability, e.g., indicated by Grades III or IV of the Graded Chronic Pain Scale, are usually transferred to the specialized consultation hour in the dental school that is held by an expert in TMD diagnosis and treatment.

All the psychosocial and physical results were finally re-evaluated by two independent and experienced operators (one operator: DC/TMD Level 3) by using the assessment scoring report form and the DC/TMD decision trees. In case of discrepancies between the scorings, an agreement was reached by discussion; these results were counted as gold examiner results.

The scores and the measured severity levels of the axis II instruments were analyzed for reliability (undergraduate students vs. gold examiner), computing intraclass correlation coefficients (ICC 2,*k*) for continuous data or kappa values for ordinal data. The reliability coefficients were interpreted as 0.75–1.00 indicating excellent, 0.60–0.74 indicating good, 0.40–0.59 indicating fair, and < 0.40 indicating poor reliability [22]. Differences between groups of patients were determined using Mann-Whitney U tests. The level of significance was set to α = 0.050. Statistics were performed with SPSS Statistics 24 (IBM, Armonk, New York, United States). The study was approved by the local ethics committee (134–11-18,042,011) and met the requirements of the Declaration of Helsinki. The participants gave their signed, informed consent.

## Results

Pilot-testing revealed that the undergraduate students encountered the greatest difficulties during the calculations of chronic pain intensity (CPI), disability score (DS), and disability points (DP). Therefore, the scoring form was modified, and the calculations of CPI were visually separated from the other calculation steps. The calculations of DS was color-coded, and arrows were included to visualize the calculation of DP (Fig. 1).

The modified form was used for field-testing with 65 undergraduate students (average age 25 ± 3 years; age range 21–36 years; 69.2% females) who examined a total of 65 patients. Comparing the gold examiner and undergraduate scoring results, the ICC values for continuous data ranged between 0.81 and 1.00, and the kappa values ranged between 0.84 and 1.00 (Table 2). While 62.9% of the undergraduate students confirmed that completing the scoring form was easy, 30.6% were undecided, and 6.5% disagreed. Of the undergraduate students, 83.0% indicated that they would at least sometimes use the scoring form in their future daily dental practice, 13.9% disagreed, and 3.1% did not respond.

**Table 2:** Reliability coefficients comparing inexperienced examiners’ and gold standard examiner’ results for axis II scoring criteria; Intraclass correlation coefficient (ICC) and 95% confidence interval

Scoring criterion	ICC	Kappa Value
Characteristic Pain Intensity (CPI)	0.81 (0.69–0.89)	
Disability Points (DP)	1.00 (0.99–1.00)	
Categories GCPS		0.84 (0.71–0.98)
Sum score PHQ-9	1.00 (1.00)	
Categories PHQ-9		1.00 (1.00)
Sum score PHQ-15	1.00 (0.99–1.00)	
Categories PHQ-15		1.00 (1.00)
Sum score GAD-7	1.00 (0.99–1.00)	
Categories GAD-7		0.97 (0.90–1.00)

*GCPS* Graded Chronic Pain Scale, *PHQ* Patient Health Questionnaire, *GAD* Generalized Anxiety Disorder

According to the DC/TMD decision trees, the gold examiner results for all the patients (*n* = 80; average age 31 ± 13 years; age range 19–73 years; 71.3% females) revealed that 21.2% received no TMD diagnosis (NoTMDdx) and 78.8% could be categorized with TMD (TMDdx). Of all the patients, 10.0% presented a pain-related disability Grade III or IV. Moderate-to-severe levels of depression were observed in 15.0%, anxiety in 7.6%, and non-specific physical symptoms in 16.3% (Table 3). No statistically significant differences were detected between the psychosocial characteristics of the two patient groups (Mann Whitney-U tests; *p* ≥ 0.063).

**Table 3:** Results of the self-report questionnaires as a percentage and number of the 80 patients presenting with TMD-associated complaints (gold standard examiner results presented) and patient group comparisons; NoTMDdx: no TMD diagnosis according to Diagnostic Criteria for Temporomandibular Disorders (DC/TMD) decision trees; TMDdx: TMD diagnosis according to DC/TMD decision trees

Instrument	Interpretation					*P*-value
GCPS (1 month)	Grade 0	Grade I	Grade II	Grade III	Grade IV	
All patients (80)	8.8 (7)	76.3 (61)	5.0 (4)	7.5 (6)	2.5 (2)	–
NoTMDdx (17)	11.8 (2)	76.5 (13)	5.9 (1)	5.9 (1)	0.0 (0)	0.523
TMDdx (63)	7.9 (5)	76.2 (48)	4.8 (3)	7.9 (5)	3.2 (2)	
PHQ-9	None	Mild	Moderate	Mod-Severe	Severe	–
All patients (80)	61.3 (49)	23.8 (19)	12.5 (10)	2.5 (2)	0.0 (0)	
NoTMDdx (17)	70.6 (12)	17.6 (3)	11.8 (2)	0.0 (0)	0.0 (0)	0.383
TMDdx (63)	58.7 (37)	25.4 (16)	12.7 (8)	3.2 (2)	0.0 (0)	
PHQ-15	None	Mild	Moderate	Severe		–
All patients (80)	42.5 (34)	41.3 (33)	13.8 (11)	2.5 (2)		
NoTMDdx (17)	64.7 (11)	23.5 (4)	11.8 (2)	0.0 (0)		0.063
TMDdx (63)	36.5 (23)	46.0 (29)	14.3 (9)	3.2 (2)		
GAD-7	None	Mild	Moderate	Severe		–
All patients (80)	72.5 (58)	20.0 (16)	6.3 (5)	1.3 (1)		
NoTMDdx (17)	70.6 (12)	23.5 (4)	5.9 (1)	0.0 (0)		0.898
TMDdx (63)	73.0 (46)	19.0 (12)	6.3 (4)	1.6 (1)		

*TMD* Temporomandibular Disorders, *GCPS* Graded Chronic Pain Scale, *PHQ* Patient Health Questionnaire, *GAD* Generalized Anxiety Disorder

## Discussion

The field-testing of the scoring form for the assessment of psychosocial characteristics revealed that it is a practicable tool for inexperienced examiners. The majority of the undergraduate students rated the collection and scoring of the data as simple, and only few examiners stated that they found the calculations inconvenient or the questionnaires too time consuming. The extent of the questionnaires might be a relevant issue in daily dental practice or dental education. Therefore, it is possible to employ the PHQ-4 as a “shortcut” screening instrument for anxiety and depression and as a substitution for PHQ-9 and GAD-7 [23]. The PHQ-4 is a four-item questionnaire comprising two questions for anxiety and depression. As validated cut-off values are available for this ultrashort questionnaire, it would be possible to create a “flag” system and implement the PHQ-4 within the psychosocial assessment scoring form [24–26]. As our dental school is specialized in the diagnosis and treatment of patients with TMD, we also have an educational focus in the field of TMD, especially for the clinical courses. Thus, many patients with TMD-associated complaints are referred from dentists in the surrounding areas for diagnosis and treatment. Therefore, we use the comprehensive assessment of psychosocial characteristics recommended by DC/TMD. Moreover, the authors believe that it improves the understanding of the complexity of psychosocial conditions. Nonetheless, students are taught about the option of using PHQ-4 for screening purposes.

The results of the scoring form can be used as a starting point for a conversation with the patient about psychosocial topics. The scoring form objectively visualizes the results, as the meaning of the “flag or traffic light system” is common, and red lights usually indicate an urgency to stop the usual behavior and, in the patient’s case, to address suspicious axis II characteristics. In our dental school, patients with “red flags” are usually transferred to the specialized consultation hour held by an expert in the diagnosis and treatment of TMD (DC/TMD Level 2 or 3). In daily dental practice, a red flag could led to referral to an expert for TMD or orofacial pain. Moreover, the psychosocial scoring assessment form can be beneficial for the diagnosis of other chronic pain conditions in the orofacial region, such as occlusal dysesthesia or burning mouth syndrome [27, 28]. However, as psychosocial education is not only important in dental education, it should be a major focus in other disciplines, since psychosocial characteristics have a high impact in, e.g., lower back pain, Parkinson’s disease, or cardiovascular diseases [29–31].

Some undergraduate students had difficulties with the GCPS scoring procedures that are not as easy to apply as those in other instruments. This phenomenon can mainly be attributed to mean value calculations, which have to be assigned to reference points. As a result of these observations, the disability score calculation area was color-coded, and arrows were inserted to simplify the scoring procedure. Digital psychosocial assessment tools and computer-based scoring approaches might help minimize the risk of errors when summarizing results, but online data protection is challenging, and the access/handling of computer-based questionnaires might be difficult for older/digitally naive people. Moreover, paper-based evaluations have the advantage that examiners have to engage themselves with every single question, which might enhance the examiner’s understanding of the psychosocial characteristics of the patient. The questionnaires can serve as guideline for patient interviews and can help examiners break down first barriers and reduce uncomfortable feelings when asking sensitive questions.

Examiners praised the free, online availability of the paper-based assessment instruments for axis II characteristics. Moreover, an advantage of the questionnaires used is their availability in various languages, since parts of the assessment can be performed in the patient’s native language, and the results can be easily interpreted for scientific purposes worldwide. Since all the questionnaires are also used in general medicine, the data gathered in studies on patients with TMD can be easily compared to the results from other disciplines.

The strengths of this study include the application of validated and reliable instruments for physical and psychosocial assessments that are available in several languages. To the best of the authors’ knowledge, the practicability of a standardized assessment protocol for psychosocial comorbidities by inexperienced examiners was investigated for the first time. However, the current study employed only a selection of the self-report questionnaires recommended by DC/TMD [13] and undergraduate students. It might be interesting to assess how dental hygienists or experienced dentists evaluate the scoring form. Nonetheless, the results obtained in this study emphasize that inexperienced examiners can easily handle the application and interpretation of the psychosocial assessment scoring report form.

## Conclusions

Within the limitations of the present study, the scoring report form for the assessment of psychosocial comorbidities of TMD patients proved to be a practicable and easy-to-use tool for inexperienced examiners. The use of the psychosocial assessment scoring form might help to improve the understanding and attitude of undergraduate students or general dentists regarding the examination of axis II characteristics, since it facilitates the evaluation of the questionnaires and easily visualizes the results as a flag system for both the operator and patient.

## Data Availability

All the data generated from this study are available from the corresponding author upon reasonable request.

## Abbreviations

CPI: Chronic pain intensity
DC/TMD: Diagnostic Criteria for Temporomandibular Disorders
DP: Disability points
DS: Disability score
JFLS: Jaw Functional Limitation Scale
GAD: Generalized Anxiety Disorder
GCPS: Graded Chronic Pain Scale
ICC: Intraclass correlation coefficients
NoTMDdx: Patients receiving no TMD diagnosis according to DC/TMD decision trees
OBC: Oral Behaviors Checklist
TMD: Temporomandibular disorders
TMDdx: Patients receiving a TMD diagnosis according to DC/TMD decision trees
PHQ: Patient Health Questionnaire
